# Rate and determinants of non-adherence to a gluten-free diet and nutritional status assessment in children and adolescents with celiac disease in a tertiary Brazilian referral center: a cross-sectional and retrospective study

**DOI:** 10.1186/s12876-018-0740-z

**Published:** 2018-01-19

**Authors:** Maraci Rodrigues, Glauce Hiromi Yonaminez, Carla Aline Satiro

**Affiliations:** 1 Department of Gastroenterology, Hospital das Clínicas, School of Medicine, University of Sao Paulo (SMUSP), Av. Dr Eneas de Carvalho Aguiar, 255, 05403-000, Sao Paulo, Brazil; 2 Department of Pediatric, Instituto da Criança, Division of Nutrition, Hospital das Clínicas, School of Medicine, University of Sao Paulo (SMUSP), Sao Paulo, Brazil

**Keywords:** Celiac disease, Nutritional status, Gluten-free diet

## Abstract

**Background:**

Compliance with a gluten-free diet (GFD) is difficult at all ages but particularly for teenagers due to social, cultural, economic, and practical pressures. The multidisciplinary team responsible for the treatment of patients with celiac disease and give support to their parents plays a special role on strengthening GFD and assessing the nutritional and physical health.

**Methods:**

A cross-sectional and retrospective study including patients under 20 years of age, with biopsy-confirmed CD, followed regularly at the Department of Pediatrics, Division of Gastroenterology, *Hospital das Clínicas*, University of Sao Paulo, School of Medicine, Sao Paulo, Brazil, were surveyed using a questionnaire and serologic test applied between November 2011 and February 2012. A retrospective chart review of these patients was performed to collect the anthropometric data along with the results of the serologic test performed at the time of diagnosis and after at least 1 year of treatment with a GFD.

**Results:**

We evaluated 35 patients aged between 2.4 and 19.9 years. Of these 68.6% were female, 88.6% had the typical form of the disease and 51.4% had other comorbidities. The mean age at diagnosis was 5.4 years. Despite dietary guidance, 20% reported non-adherence to the diet. Most children recovered the weight and height deficit after 5 years of treatment, and in some children, excessive weight gain became a concern.

**Conclusion:**

The majority of transgressions occurred intentionally at home or at parties. There was a risk of excessive weight gain, especially in the first two years of treatment. More alternatives and easier access to low cost gluten-free foods, increasing the discussion about the benefits of adhering to a GFD among patients, families, and the general population, besides the acquisition of self-management skills, are crucial to fostering independent children and adolescents who have the knowledge and tools to manage life with CD.

**Electronic supplementary material:**

The online version of this article (10.1186/s12876-018-0740-z) contains supplementary material, which is available to authorized users.

## Background

Celiac disease is now defined as a genetically predisposed systemic autoimmune condition characterized by the presence of a variable combination of gluten-dependent enteropathy, other clinical manifestations, and CD-specific antibodies such as anti-tissue transglutaminase antibodies 2 (TG2-IgA and TG2-IgG) and anti-endomysial antibodies (EMAs) [1] . The disease occurs in individuals carrying the HLA class II DQ2 or DQ8 allelic variants haplotype and is characterized by an inflammatory enteropathy with various degrees of severity, and a wide range of gastrointestinal and extraintestinal symptoms and signs [1]. CD can reach far beyond the intestine and has been associated with many non-gastrointestinal signs and symptoms such as dermatitis herpetiformis, short stature, osteoporosis, iron deficiency anemia, arthritis, headaches, fatigue, liver function abnormalities, myalgias, adverse pregnancy outcomes, tooth enamel defects, and others [2]. A strong association with autoimmune conditions such as type 1 diabetes mellitus has been observed, and it has been linked with some congenital disorders, such as IgA deficiency and Down syndrome [2] . This pathology is considered a major public health problem because of its high prevalence worldwide. Studies from the United States and Europe estimated that the mean prevalence of CD in the general population is approximately 1%, with some regional differences. A similar disease prevalence has been found in other countries mostly populated by European descendants [3].

In Brazil, the prevalence of biopsy-confirmed CD in apparently healthy volunteer blood donors ranged from 1:214 to 1:286 in Sao Paulo city [4, 5] 1:273 in Sao Paulo countryside [6], 1:417 in southern Brazil [7] and 1:681 in Brasilia [8] . These preliminary results support the view that CD is not a rare disease in Brazil.

Currently, the treatment of CD is based on lifelong exclusion of wheat, rye and barley [9], oats (because in Brazil oats may be contaminated with gluten), and other gluten-containing food products from the diet.

Adherence to a GFD is crucial not only for intestinal mucosal recovery and alleviation of symptoms, but also for the prevention of complications such as anemia, osteoporosis fractures and small-bowel lymphoma [9].

Compliance with a GFD is difficult at all ages but particularly for teenagers due to social, cultural, economic, and practical pressures. At this age group, the diet may be a psychological and social challenge. Occasions such as birthday parties, sleepovers, eating out, and even snack time at school can be difficult to be managed. A survey among college students with CD revealed that they were motivated to adhere to the diet but they experienced challenges related to dining services and social meetings [10].

The strategies required for a GFD to be successful must include education for the patient, family and all those involved with the patient (e.g. healthcare team and school) regarding product ingredients, labeling all gluten-free flours, having snacks on hand at school or work, and bringing gluten-free foods to social events [11]. With regard to parental knowledge, there is the need for clinicians and healthcare professionals to provide better education and more efficient counseling about gluten-free products for parents [12].

Although evidence-based recommendations for follow-up of pediatric patients with CD have not yet been established, some experts advise a yearly follow-up visit and monitoring of dietary compliance, with evaluation of the improvement in symptoms and signs, and analysis for biochemical and serologic markers of CD and screening for associated autoimmune thyroiditis [13].

Therefore, the aims of this study were to examine the rate and determinants of non-adherence to a GFD, as well as the dietary habits and nutritional status of children and adolescents diagnosed with CD at a tertiary referral center.

## Methods

This was a cross-sectional and retrospective study, and included 35 patients with CD who attended a medical consultation, conducted in the Pediatric Gastroenterology Outpatient Clinic of the Department of Pediatrics, SMUSP, Sao Paulo, Brazil, between November 2011 and February 2012. These patients were children and adolescents with confirmed diagnosis according to the CD consensus of 2012 [14]:

1.Typical or atypical manifestations or belonging to a group at risk for CD;

2.Positive serology (TG2 IgA, EMAs, or TG2 IgG if hypogammaglobulinemia IgA was diagnosed) or presence of human histocompatibility antigen genes (HLA) class II DQ2 or DQ8 allelic variants;

3. Confirmation of the diagnosis by duodenal biopsy, from the bulb (at least one biopsy) and from the second and third portion of the duodenum (at least four biopsies), and grading according to marsh-Oberhuber [15].

The celiac follow-up visits were defined as attending a medical appointment that addressed CD and documented symptoms associated with CD, GFD compliance, and/or CD serologies.

The medical team included pediatric gastroenterologists, nutritionists and social assistants to assess each child and teenage carrier of CD, and their parents, in relation to symptoms, nutritional status, education, adherence and social difficulties associated with adhering to a GFD.

Patients younger than 1 year or older than 20 years and those who did not attend the consultation and/or with whom contact was not possible because of obsolete phone numbers were excluded. Two patients did not agree to participate in the study.

Data were collected using a questionnaire constructed for this study comprising two open questions on social events and difficulties with the GFD, and closed questions about food routine (home, school, parties, travel), awareness, adherence and difficulties regarding the GFD.

This questionnaire was applied by trained professionals. Parents of patients younger than 13 years of age were instructed to complete the survey with their child. Adolescents older than 13 years of age were asked to complete the survey themselves, during the appointment. The questionnaire are presented in Additional file 1.

Additional information was obtained from medical records, including associated diseases, laboratory tests and anthropometric assessment at the time of diagnosis, 1, 2 and 5 years after diagnosis.

The anthropometric data of weight and height were measured during the medical consultation, on the day of application of the questionnaire. Weight was measured on digital scales, with accuracy to 100 g, while the patient was barefoot and in his underwear. Height was measured using a horizontal stadiometer in children under 2 years of age and a vertical stadiometer in children above this age.

The anthropometric data of weight, height and body mass index (BMI) were expressed as Z scores using the World Health Organization (WHO) multicentre growth reference study group [16].

The laboratory tests were performed according to the reference laboratory of SMUSP.

### Ethics approval and consent to participate

This project was approved by the Research Ethics Committee (REC) and the Ethics Committee for review of research projects (CAPPesq) under the number 829/11.

Patients (above 16 years of age), parents or legal guardians (in the case of children under 16) only participated in the study after signing the informed consent, which provided information on the goals of the study and other relevant information.

Permission to conduct the study was sought from relevant ethical committees at Department of Pediatric, Instituto da Criança, Hospital das Clinicas, São Paulo, Brazil. All patients were entered into the study after a written informed consent, either given by the patients themselves or their guardians in the case where patients were not able to.

### Statistics

All data were coded in numerical form. The data tabulation and analysis were performed using Microsoft Excel, Epi Info, version 6.04, and the Statistical Package for the Social Sciences, version 13.0 (SPSS Inc. Chicago, IL, USA).

Fisher’s exact test was the statistical test used to determine if there were nonrandom associations between two categorical variables, and McNemar’s test was used to compare paired proportions. Significance was defined as *P* < 0.05.

## Results

### Characterization of the population

Were included a total of 35 patients, aged from 2.4 to 19.9 years (mean: 10.77, standard deviation: 4.16 and median: 11 years); 68.6% were female. In most cases (51.35%), the questionnaires were answered by the patient’s mother (Table 1).

**Table 1:** Characteristics of the population

Features	N	%
Gender		
Boys	24	68.6
Girls	11	31.4
Age group		
2–5 years	5	14.3
5–12 years	15	42.9
> 12 < 19.9 years	15	42.9
Manifestation of the disease		
Typical	31	88.6
Atypical	4	11.4
Age at diagnosis		
0–2 years	8	22.9
2–5 years	9	25.7
5–12 years	14	40
> 12 years	0	0
No response	4	11.4
Time of disease		
0–2 years	8	22.9
2–4 years	5	14.3
4–6 years	4	11.4
6–8 years	4	11.4
8–10 years	3	8.6
> 10 years	6	17.1
No response	5	14.3
Monthly income		
Up to 1 minimum wage	9	25.7
Up to 2 minimum wage	13	37.1
Up to 3 minimum wage	7	20
> 3 minimum wage	6	17.1
Number of residents in the house		
1–3	8	22.9
4–6	25	71.4
> 7	1	2.9
No response	1	2.9

Patients or their family considered their health to be good, very good or great (91.4%) when compared to other people of the same age without the disease.

About 51.4% of respondents had another disease besides CD, the most cited were: diabetes mellitus type 1 23% (*n* = 8), asthma 14% (*n* = 5), and rhinitis and epilepsy 11.5% (both with four cases).

The majority of patients (91.4%) did not have knowledge about CD before diagnosis, and 93.8% reported that they had been suffering from symptoms characteristic of the disease (mainly diarrhea) for about 23.6 months before diagnosis (DP: 24 months).

### Dietary treatment

Most patients consumed meat, milk, fruit and vegetables on a daily basis, as per Table 2.

**Table 2:** Food profile of patients with celiac disease

	Meat	Milk	Fruit	Greens and vegetables
Daily	62.9%	91.4%	62.9%	54.3%
2 to 4 times a week	37.1%	8.6%	31.4%	37.1%
Does not consume	–		5.7%	8.6%

When at school, 48% (*n* = 24) of the patients brought a snack from home, 18% (*n* = 9) bought lunch. When the option was to purchase food at school, the favorites were: cheese bread (25.7%),wich is very popular in Brazil and it is made with cassava flour and cheese, snacks (22.9%), soda (14.3%); and 69.2% did not receive GFD at school.

All patients were instructed to follow a restricted diet, but 20% (*n* = 7) reported that they did not avoid gluten at all times. The latter presented a positive EMAs result.

Factors cited as justifications for transgressions were the high cost of a GFD (*n* = 2; 15.4%), the low acceptance of these foods (n = 2; 15.4%), lack of choice (*n* = 1; 7.7%), visiting friends (n = 1; 7.7%), and voluntary choice (*n* = 7; 53.8%).

Transgressions occurred at parties (*n* = 12; 23.5%), home (*n* = 11; 21.6%), school (*n* = 10; 19.6%), while shopping (*n* = 8; 15.7%), with friends (n = 7; 13.7%), and while traveling (*n* = 3; 5.9%). A total of 51.9% claimed that the transgressions were intentional and 63.3% reported having symptoms after the transgressions, primarily diarrhea (41.4%) and abdominal pain (41.4%). The frequency of flatulence was higher in the group that did not strictly adhere to the diet (*p* = 0.083).

When asked about their behavior at parties and social events, the majority reported taking food from home to avoid eating food containing gluten, while 62.8% of patients stated that they never attended events due to their disease. See Table 3.

**Table 3:** Behavior of patients with celiac disease at parties and social events

Behavior at parties	%
Takes food from home	65.7%
Ashamed and does not attend	11.4%
Only eats permitted foods	5.7%
Does not eat	8.6%
Infringes the GFD	5.7%

Gluten-free recipes were prepared by 88.6% of the patients and/or their guardians, and 41.9% prepared special recipes three times a week. The main recipes with satisfactory sensory characteristics, according to the patients, were: cakes (80%) and pies (20%). Bread (20%) and pancakes (8.57%) were considered to have less satisfactory sensory characteristics. The flours mostly used as a substitute for gluten were: corn starch, corn flour and potato starch. All patients reported buying gluten-free foods in conventional supermarkets (*n* = 30) and specialist shops (*n* = 26).

Meals outside the home were eaten a few times per month by 45.7% of the patients, mainly in restaurants and coffee shops.

When asked about difficulties related to adhering to the treatment of celiac disease, 37.1% had no difficulty, 25.7% little difficulty, and 20% average difficulty, while 17.1% found it very difficult. The majority of patients (74.3%) were able to talk with ease about celiac disease, but a significant percentage, either disliked or were ashamed of talking about it, 17.1% and 14.3% respectively. All claimed to know about the risks of non-adherence to the diet and to know that dietary treatment would be lifelong.

There was no association between diet transgression and gender form of presentation of the disease, eating habits, behaviors in relation to food in school and social events or knowledge about the disease.

### Nutritional evaluation

Out of the 35 patients, 27 had anthropometric data of diagnosis, enabling an assessment of nutritional evolution. The remaining patients were diagnosed in another service or did not have this data in their chart.

At the time of diagnosis 4 children were classified as having a nutritional deficit according to their BMI/age, either being underweight or extremely underweight but this index improved considerably during treatment; no child had a weight deficit at the end of the study.

In the same way, when the children were evaluated in accordance with their stature/age i.e. the number of children with a height deficit decreased during treatment.

The number of children who were overweight or obese increased throughout the follow-up of these patients. See Tables 4 and 5.

**Table 4:** Nutritional status of patients with celiac disease according to the body mass index for age (BMI/age)

	At time of diagnosis	1 year	2 years	5 years	Current
Extremely underweight	2	0	0	0	0
Underweight	2	0	1	0	0
Eutrophy	18	16	8	9	19
Risk of overweight	2	4	3	0	2
Overweight	2	3	2	0	4
Obesity	1	2	2	2	2
Total	27	25	16	11	27

**Table 5:** Stature for age of patients with celiac disease at diagnosis and over time

	At time of diagnosis	1 year	2 years	5 years	Current
Very short stature for age	4	3	2	1	1
Short stature for age	5	4	2	1	3
Appropriate stature for age	18	18	12	9	22
Total	27	25	16	11	26

There was no difference in the nutritional parameters (BMI/age and stature/age) between patients who reported transgression of the diet and those who demonstrated adherence to treatment.

It was also assessed the laboratory tests of these patients, and the main change was the EMAs, with about 28 patients with a positive result. The results of this blood tests were not statistically different over time.

## Discussion

This study demonstrated that 20% of evaluated patients did not adhere completely to the GFD. The reasons for transgressions were related to the GF products (high cost, low palatability and lack of availability), social events with friends and voluntary choice. Surprisingly, the nutritional parameters were not influenced by the adherence to the diet. Probably, the transgression of the GFD was not high enough to compromise the weight gain and growth of the patients.

Our sample was small compared with some previous studies [12, 17–23], but larger than in others [24, 25], because our study was conducted in a single center and included only patients with regular outpatient gastroenterologist visits in the period between November 2011 and February 2012.

The majority of our patients were women (68.6%), which was consistent with other studies, in which a slight predominance of females was observed [12, 17–20, 22, 24, 26].

The mean age of the 35 children included in this study was 10.77 years and the median was 11 years; they were older than in some studies [17, 22, 25] and younger than in other studies [12, 19, 20, 24].

In our study, the genre and clinical presentation of celiac disease at diagnosis had no effect on adherence. This is consistent with several other studies [12, 18, 22], but contrasts with others [27], in which adherence was better in girls than in boys.

In our study, the adherence of adolescents (older than 13 years of age) to a GFD was particularly low while dining at restaurants, maybe due to lack of easy availability of GF foods outside the home or peer pressure when dining out, as in other studies [12, 18, 19, 21]. In the Ljungman and Myrdal study [27], they stratified the adolescents’ ages and observed that the adherence was better in younger adolescents aged 12 to 14 years old than in older adolescents aged 15 to 17 years old, probably because the latter group had greater social exposure than the former.

In our study the majority (93.8%) of patients reported that they had suffered from symptoms of the disease for about 23.6 months before diagnosis (DP: 24 months), similar to the time span reported in another study [12], and longer than in others [17, 18, 22, 26].

In our series, most of the children had typical symptoms of CD (88.6%), which was consistent with the study by Charalampopoulos et al. [12] but higher than in other studies [17, 18, 22, 26]. Our findings also contrast with those of Munoz et al. [24], who reported that children with atypical symptoms before diagnosis and patients with screening diagnosis by serologic testing had a higher rate of transgression than children with typical symptoms.

Other studies reported similar reasons, regarding to the availability of GF foods and costs as the most significant barriers to adherence to a GFD [24, 28].

In contrast as described previously [18], in our study there was not an association between non-adherence and environment. Our study also agree with others studies [19, 29] that most CD patients on a GFD, specially teenagers, found it easier to adhere to the diet at home and school than when out in social situations. Our study found that the most transgressions did not occur only at friends´ house and birthday parties, but also at home and school, as reported by Munoz et al. [24].

In our patients, as well as in other study [30], when asked about the behavior at parties and social events, the majority reported that successful diet adherence strategies included planning ahead and taking their own food to social events to avoid meals containing gluten.

Most patients in our study found the GFD relatively easy to follow, were comfortable talking about their disease and realized that they would need to follow a GFD for entire life. This results differs from that reported by Bravo & Munoz [24] reported that 70% found the diet hard to follow, 55% had difficulty following the diet, 42.8% were not sure what they could eat, and 51.5% reported that the GFD was causing financial distress in the family.

In respect of involuntary transgression, it is worth mentioning the possibility of occurring with the acquisition of cheese bread outside the home (cited by nine patients) as this may be contaminated with other gluten foods produced and served alongside the bread. In addition, despite reporting adherence to the diet, the positive EMAs results in 28/35 patients indicate unintentional transgression.

Error or mistake was also cited in the literature as a reason for transgressions. In Brazil the law requires that all processed products show the information “contains” or “does not contain gluten”, and even with disbelief in some information on the label, it facilitates the purchase of industrial products. However, errors or mistakes often happen with ready-to-eat products, consumed in cafes, restaurants and while traveling, so it is important that patients and families are able to cope with these situations. In this study, 48.1% of patients report that the transgression was not intentional, but rather due to the lack of adequate information about the preparation of food.

Similar to our results, several studies have found that patients found it particularly difficult to adhere to a GFD when dining out or at social events, due to variability in the availability of GFD foods or cross-contamination with gluten.

As our study, the higher price of gluten free products is a point of great concern, and several studies from North America and Europe have demonstrated that the cost of GFD food products is significantly higher than that of gluten-containing foods [31–33].

Similar to our results, Tapsas et al. [20] reported different symptoms after incidental intake of gluten, including abdominal pain (64.8%), vomiting (33.3%), and diarrhea (24.1%). They found that in two-thirds of the children in their study, symptoms were noticed soon after consumption, that is, during the first 3 h. No correlation was found between the amount of accidental gluten intake and the time of symptom onset. Some patients, however, experienced symptoms later, that is, after more than 24 h.

Dietary adherence has been the subject of several investigations in pediatric CD and these studies have varied with respect to sample size and method of assessment of adherence. Measures used to assess adherence included self-report, interview, dietary record, and biossay methods, and each measure has its strengths and limitations. Available methods are insufficiently accurate to identify occasional gluten exposure that may cause intestinal mucosal damage. Serological tests are highly sensitive and specific for diagnosis, but do not predict recovery and are not useful for follow-up. Serial endoscopy is invasive and impractical for frequent monitoring, and dietary interview can be subjective [10]. The detection of gluten immunogenic peptides in feces and urine as new non-invasive biomarkers of gluten intake has been suggested, but requires further investigation [34].

In our study, the overall rate of adherence was 80%, as measured by questionnaire and serologic testing. Another Brazilian study conducted by ACELBRA (Brazilian Celiac Association) [35] found that 29.5% of patients reported transgressions in their diet, a higher percentage than the present study. The rates of adherence to GFD in the literature vary from 39% to 79%, depending on factors including self-reporting, laboratory testing and prediagnosis symptoms [12, 18, 19, 36–39]. Some of the studies, despite the small sample, illustrated that non-adherence to GFD is related to poorer bone health [36, 37], shorter stature [37]. Parent report adherence via interview was higher than bioassay data [38] tTG antibodies were significantly more sensitive at identifying occasional non-adherence than EMAs data [39]. Younger age at diagnosis, currently being a teenager, and current symptoms were associated with non-adherence [18]. There was a relationship between compliance, children’s age and perceived parental knowledge [12]. Younger age at diagnosis and shorter duration since the diagnosis were associated with a better adherence rate [22].

Our study did not compare the potential difference in diet adherence when questionnaires were filled in by adolescents or by their parents, or if the CD education program for parents and caregivers of celiac patients improved adherence to GFD rate. In our service all the family and responsible for celiac patients receive education about CD routinely.

It has been reported that parents could give us a false picture of the children’s compliance with the GFD [12, 40] On the other hand, other authors reported that the perceived parental knowledge was also independently and significantly associated with dietary compliance [12].

We would like to strengthen the importance of the education on CD and the adherence of a GFD not only to the pediatric patients but also to the family and all those involved with the patient to achieve the adherence to a strict lifelong gluten-free diet (GFD).

In our study, children and adolescents recovered their nutritional status after starting treatment, with a significant increase in the proportion of overweight patients over the years. There was no statistical relationship between adherence to treatment and nutritional status, which result was in agreement with other study [41]. They also reported that children who did not adhere to the GFD had lower values of the Z-score of stature/age in comparison with children who adhered to the GFD; 5.9% of children were overweight, which was lower than found in our study.

Other study which reported nutritional status [26], found that almost 19% of patients had an elevated BMI at diagnosis (12.6% overweight, 6% obese) and 74.5% presented with a normal BMI. Seventy-five percent of patients with an elevated BMI at diagnosis decreased their BMI Z-score significantly after adherence to a GFD, normalizing it in 44% of cases. Of patients with a normal BMI at diagnosis, weight Z-scores increased significantly after treatment, and 13% became overweight. In this study, a GFD may have a beneficial effect upon the BMI of overweight and obese children with CD. Other aspect about nutrition and CD was observed in other study [25] which reported that children with CD had high intakes of fiber, and foods with a high glycemic index, and glycemic load, and lower folate content than controls. This has implications for dietary counseling in this population.

The limitations of our study were the sample size and the lack of complete data regarding nutritional evolution, which may have interfered in the absence of association between transgression and other variables, including nutritional status. The retrospective analysis of medical records and questionnaires may involve registration and memory bias, respectively, which may influence the accuracy of the results.

## Conclusion

In this study, a fifth of the patients was voluntarily transgressing the GFD, mainly because of the high cost, lack of availability and poor palatability related to the diet. Possibly, part of the children in sample is also involuntarily eating gluten, as observed by the positivity of the serologic tests. There was a catch-up growth in patients with nutritional deficit and a risk of excessive weight gain during follow-up. The early diagnosis and continuous education, with a multidisciplinary clinical care model, is essential to promote adherence to the diet.

## Additional file

Additional file 1: Questionnaire for assessment of adherence to the treatment of patients with Celiac Disease. Questionnaire constructed for this study comprising two open questions on social events and difficulties with the GFD, and closed questions about food routine, awareness, adherence and difficulties regarding the GFD. (DOC 75 kb)

## Abbreviations

ACELBRA: Brazilian Celiac Association
BMI: Body mass index
CAPPesq: Ethics Committee for review of research projects
CD: Celiac disease
EMAs: Anti-endomysial antibodies
GFD: Gluten-free diet
HLA: Human histocompatibility antigen genes
REC: Research Ethics Committee
SMUSP: School of Medicine, University of Sao Paulo
SPSS: Statistical Package for the Social Sciences
TG2-IgA: Transglutaminase antibodies 2
WHO: World Health Organization
