# Correction to: Rate and determinants of non-adherence to a gluten-free diet and nutritional status assessment in children and adolescents with celiac disease in a tertiary Brazilian referral center: a cross-sectional and retrospective study

**DOI:** 10.1186/s12876-018-0757-3

**Published:** 2018-02-28

**Authors:** Maraci Rodrigues, Glauce Hiromi Yonamine, Carla Aline Fernandes Satiro

**Affiliations:** 1 Department of Gastroenterology, Hospital das Clínicas, School of Medicine, University of Sao Paulo (SMUSP), Av. Dr. Eneas de Carvalho Aguiar, 255, 05403–000, Sao Paulo, Brazil; 2 Department of Pediatric, Instituto da Criança, Division of Nutrition, Hospital das Clínicas, School of Medicine, University of Sao Paulo (SMUSP), Sao Paulo, Brazil

## Correction

Unfortunately, after publication of this article [1], it was noticed that the names of the second and third authors were incorrectly displayed, respectively, as Glauce Hiromi Yonaminez and Carla Aline Satiro. The correct names are Glauce Hiromi Yonamine and Carla Aline Fernandes Satiro and can be seen in the corrected author list above. The original article has also been updated to correct this error.
