# Supplementary material for: Rate and determinants of non-adherence to a gluten-free diet and nutritional status assessment in children and adolescents with celiac disease in a tertiary Brazilian referral center: a cross-sectional and retrospective study
Source: BMC Gastroenterol. 2018 Jan 19;18:15. doi: 10.1186/s12876-018-0740-z (PMC5775619; doi:10.1186/s12876-018-0740-z)
Supplement: Additional file 1: — Questionnaire for assessment of adherence to the treatment of patients with Celiac Disease. Questionnaire constructed for this study comprising two open questions on social events and difficulties with the GFD, and closed questions about food routine, awareness, adherence and difficulties regarding the GFD. (DOC 75 kb) [file 12876_2018_740_MOESM1_ESM.doc]

**Questionnaire for assessment of adherence to the treatment of patients with Celiac Disease**

Who is answering the questionnaire: _______________________________________

Name (patient):________________________________________________________

Gender: (   ) Female             (   ) Male

Date of birth: ____ / ____ / ____                 Age:_____

How long has the diagnosis of Celiac Disease been established?

_______ years ______ months

**1.-**How many people live in the house? _______

**2 -** What is the family's monthly income?

(  ) Up to 1 minimum wage (R$ 545,00)

(  ) Up to 2 minimum wages (R$ 1090,00)

(  ) Up to 3 minimum wages (R$ 1635,00)

(  ) Up to 4 minimum wages (R$ 2180,00)

(  ) More than 4 minimum wages (R$ 2180,00)

**3 -** Compared to other people of the same age, the patient's health is considered by him and his family:

(  ) Great

(  ) Very Good

(  ) Good

(  ) Average

(  ) Bad

**4 -** Are there other people with celiac disease in the family? If so, answer:

How many people: ________

Age: ______             Gender: _____               Year of diagnosis: _____

Follows a gluten-free diet: ( ) Yes   ( ) No

Type of kinship with the patient:___________________________

**5 -** Before diagnosis:

Did you know what was celiac disease? ( ) Yes ( ) No

Did you have symptoms of celiac disease? ( ) Yes ( ) No

If so, for how long until the diagnosis? ____ years ____ months

**6-** How often does the patient eat the food below:

- Meat and eggs:        (  ) Daily      (   ) 2 to 4 times a week   (   ) Non-consumption

- Dairy:       (  ) Daily      (   ) 2 to 4 times a week   (   ) Non-consumption

- Fruits:                       (  ) Daily      (   ) 2 to 4 times a week   (   ) Non-consumption

- Vegetables: (  ) Daily      (   ) 2 to 4 times a week   (   ) Non-consumption

**7 -** As for school snacks: (you can check more than one option)

( ) Bring snack from home

( ) Purchase at school

( ) The school provides meals

( ) Eat friends’ snack

**8-** If the school provides, is the meal gluten-free?
( ) Yes ( ) No

**9 -** If you buy the snack at school, which foods do you buy?

__________________________________________________________________________________________________________________________________________

**10 –**Does the patient always avoid all foods with gluten? (  ) Yes      (  ) No

**11 -** If no, what are the reasons why you consume foods with gluten: (you can select more than one option)

( ) own choice

( ) lack of alternative

( ) cost of gluten-free food

( ) does not like gluten-free food

( ) inadvertence (without information that the food contains gluten)

( ) Others: ___________________________________________________________

**12 –** In which places / situations have occurred transgressions to the diet? (you can check more than one option)

( ) At home

( ) House of friends and family

( ) School

( ) Parties

( ) Travels

( ) Purchase of products in bakeries, restaurants

( ) Others: ___________________________________________________________

**13** - Have the transgressions in these places / situations been intentional?
(  )Yes (  ) No

**14**- When transgressions occur, does the patient have symptoms? (  ) Yes    (  ) No

If yes, which ones:

( ) Diarrhea

( ) Constipation

( ) Distension / abdominal pain

( ) Nausea / vomiting

**15-** After the diagnosis, how often the patient has symptoms described in the table below:

|  | Daily | 2 to 4x per week | Once a week | 3x per month | Never |
| --- | --- | --- | --- | --- | --- |
| Diarrhea / loose stools |  |  |  |  |  |
| Constipation |  |  |  |  |  |
| Flatulence |  |  |  |  |  |
| Abdominal pain |  |  |  |  |  |
| Abdominal distension |  |  |  |  |  |
| Nausea / vomiting |  |  |  |  |  |
| Inapetence |  |  |  |  |  |
| Nervousness / irritability |  |  |  |  |  |
| Edema |  |  |  |  |  |

**16** - Does the patient or their family make gluten-free recipes? (  ) Yes    (  ) No

If so, how often?

( ) 3x per week ( ) 1x per week

( ) 3x per month ( ) 1x per month

( ) Rarely ( ) Never

**17** - What recipes did you do and succeeded?

_________________________________________________________________________________________________________________________________________

**18**- What recipes did you do and did not succeed? Explain why.

__________________________________________________________________________________________________________________________________________

**19** - Do you buy gluten-free food ? (  ) Yes    (  ) No

If yes, how often do you buy gluten-free foods at the shopping facilities listed below:

|  | Every Week | Sometimes in the month | 1x per month | Sometimes in the year | Never |
| --- | --- | --- | --- | --- | --- |
| Supermarket |  |  |  |  |  |
| Special Food Stores |  |  |  |  |  |
| Stores from the other cities |  |  |  |  |  |
| Others |  |  |  |  |  |

**20 –** Regarding the gluten-free foods that the patient has already tried, what is the opinion concerning:

Flavor: ( ) Great ( ) Good ( ) Bad

Texture: ( ) Great ( ) Good ( ) Bad

Variety: ( ) Great ( ) Good ( ) Bad

Price: ( ) Good ( ) Good ( ) Bad

Information on the label: ( ) Good ( ) Good ( ) Bad

**21** - For each of the following gluten-free products, first indicate how often you consume, and then whether you cook your food or purchase ready for consumption:

|  | **Frequency of consumption** | | | | | **Cook / Purchase** | | |
| --- | --- | --- | --- | --- | --- | --- | --- | --- |
|  | Daily | Weekly | Monthly | Annually | Never | Cook | Purchase | Cook and Purchase |
| Breads |  |  |  |  |  |  |  |  |
| Pastas |  |  |  |  |  |  |  |  |
| Cookies |  |  |  |  |  |  |  |  |
| Toast |  |  |  |  |  |  |  |  |
| Cakes |  |  |  |  |  |  |  |  |
| Cereals |  |  |  |  |  |  |  |  |

**22 -** Indicate which substitute is most used as a substitute for gluten:

( ) Rice flour ( ) Corn flour

( ) Soybean flour ( ) Tapioca gum flour

( ) Buckwheat flour ( ) Arrowroot flour

( ) Chickpea flour ( ) Cassava flour

( ) Rice starch ( ) Corn starch

( ) Gluten-free wheat starch ( ) Corn semolina

( ) Potato starch ( ) Potato starch

**23 -** How often do you read the labels of the industrialized products you buy?
( ) Frequently ( ) Rarely ( ) Never

**24 -** How often do you dine out?

( ) Daily ( ) Sometimes in the week

( ) Sometimes in the month ( ) Sometimes in the year ( ) Never

**25 -** When you dine out, you usually choose:

(  ) Snack bars (  ) Restaurants (  ) Bakeries

(  ) Cafe    (  ) Others

**26 -**  To Talk about the subject of being celiac in public:
( ) Does not like to talk about it

( ) Feel ashamed to talk about it

( ) Speaks freely and passes all possible information to people with the purpose of spreading information about the disease

**27 -** Are the patient and family members interested in new information on celiac disease (newspapers, magazines, internet, conferences)? (  ) Yes    (  ) No

**28 -** Does the patient and the family have information on the main health risks when the gluten-free diet is not completely followed? (  ) Yes    (  ) No

**29 -** Does the patient and the family have information that the gluten-free diet for the celiac patient is for the rest of life? (  ) Yes    (  ) No

**30-** How the patient behaves at parties, school, social environment:

( ) He/she brings food from home

( ) Feel ashamed and do not go to parties, playgrounds, parks

**31**- Has the patient ever stopped going to any social event because of celiac disease?

(  ) Yes          (  ) No

Why? _____________________________________________________________

**32-** From what age the celiac patient acquired the ability to identify the gluten-free diet:

( ) After literacy

( ) Even before literacy

**33 -** Who are the family members and caregivers that are aware of the celiac patient’s gluten-free diet (understands what celiac disease is, knows about the exclusion diet, has the ability to offer the correct diet):

( ) only the mother

( ) the parents

( ) parents, grandparents

( ) parents, grandparents, caregivers

( ) parents, grandparents, caregivers, school

**34 -** In general, do you (patient) have difficulties in following a gluten-free diet?

( ) No difficulty ( ) Little difficulty

( ) Average difficulty ( ) Very difficulty

Explain:
______________________________________________________________________________________________________________________________________________________________________________________________________________________________________________________________________________________________________________________________________________________________________________________________________________________________
